# *Salmonella* spp. and *Campylobacter* spp. in poultry feces and carcasses in Ouagadougou, Burkina Faso

**DOI:** 10.1002/fsn3.725

**Published:** 2018-07-16

**Authors:** Assèta Kagambèga, Alexandre Thibodeau, Valentina Trinetta, Daniel K. Soro, Florent N. Sama, Évariste Bako, Caroline S. Bouda, Aïssata Wereme N’Diaye, Philippe Fravalo, Nicolas Barro

**Affiliations:** ^1^ Laboratoire de Biologie Moléculaire, d’épidémiologie et de surveillance des bactéries et virus transmissibles par les aliments (LaBESTA)/Ecole Doctorale Sciences et Technologies (EDST)/Université Ouaga I Professeur Joseph KI‐ZERBO Ouagadougou 03 Burkina Faso; ^2^ Institut Des Sciences (IDS) Ouagadougou 01 Burkina Faso; ^3^ Veterinary Medicine Faculty Department of Pathology and Microbiology NSERC Industrial Research Chair in Meat‐Safety (CRSV) University of Montreal Saint‐Hyacinthe QC Canada; ^4^ Food Science Institute Kansas State University Manhattan Kansas; ^5^ Institut national de l'environnement et de recherches agricoles CNRST/INERA/CREAF Ouagadougou 01 Burkina Faso

**Keywords:** *Campylobacter*, carcasses, feces, poultry, *Salmonella*

## Abstract

The importance of *Salmonella* and *Campylobacter* as foodborne pathogens is well recognized worldwide. Poultry and poultry products are commonly considered as the major vehicles of *Salmonella* and *Campylobacter* infection in humans. The aim of this study was to investigate the hygienic status of poultry facilities and determine the prevalence of *Salmonella* and *Campylobacter* in slaughtered poultry feces and carcasses in four different markets in Ouagadougou, capital city of Burkina Faso. A total of 103 poultry feces and 20 carcasses were analyzed using microbiological standard methods. Among the 103 fecal samples, 70 were positive for *Campylobacter* ssp (67.96%) and 54 for *Salmonella* ssp (52.42%). The hippurate hydrolysis test revealed that among the 70 *Campylobacter* strains isolated from feces, 49 were *C. jejuni* (70%) and 21 were *C. coli* (30%). From the 20 carcasses analyzed, 18 were contaminated with *Salmonella* (90%) and 10 with *Campylobacter* ssp (50%). Among the 10 *Campylobacter* ssp samples isolated from poultry carcasses, all were identified as *C*. *jejuni* using the API CAMPY system and the hippurate hydrolysis test. The assessment of markets hygienic practices for production, transportation, display, and vending of meat revealed unhygienic conditions. To complete the observation of unhygienic practices, we have sampled chicken‐washing solution from the study sites and microbiological analysis of these samples revealed the presence of *Salmonella* spp in 100% of the samples. This study highlighted that poultry products on sale in Ouagadougou are highly contaminated with *Salmonella* and *Campylobacter*. To the best of our knowledge, this is the first report describing *Campylobacter* presence in the poultry industry of Burkina Faso. Our findings might help to better understand the epidemiology of *Salmonella* and *Campylobacte*r.

## 1 INTRODUCTION

Diarrheal disease is a serious health problem that causes high rates of morbidity and mortality in developing countries (Ayed, 2014). These diseases are due to the lack of hygiene and sanitation and have serious social and economic consequences that affect both individual and collective development. In Burkina Faso, diarrheal diseases constitute the second leading cause of consultation in health centers after respiratory infections (Ministère de la santé, 2016). *Salmonella* and *Campylobacter* are the main pathogens implicated in these diarrheal diseases worldwide (Mir, Kashyap, & Maherchandani, 2015). Since the early 1990s, *Salmonella* strains have played an important role in infectious diseases. They are responsible for a large number of food poisoning infections, and recently, the appearance of multiresistant strains has increased concern (Kagambèga, Lienemann, Frye, Barro, & Haukka, 2018). *Campylobacter* strains have been recognized as a major cause of bacterial gastroenteritis in humans since 1970 and are responsible for 400–500 million cases of diarrhea each year worldwide (Ruiz‐Palacios, 2007). In developing countries, the incidence among children under five years old is estimated as 40,000 cases per 100,000 per year (Oberhelmanand & Taylor, 2000). According to the World Health Organization, this incidence is underestimated (World Health Organization, 2001). However, the sources and transmission routes of *Campylobacter* and *Salmonella* in developing countries are poorly understood due to the lack of coordinated national epidemiological surveillance systems (Kagambèga, Barro, Traoré, Siitonen, & Haukka, 2012; Kariuki et al., 2006). Poultry is one of the principal asymptomatic carriers of *Campylobacter* and *Salmonella,* and the process of removing the gastrointestinal tract during slaughtering is regarded as one of the most important sources of carcass and organ contamination with these pathogens (Mir et al., 2015; Zhang et al., 2016). Cross‐contamination may occur during the preparation of these carcasses, increasing the risk of contamination for the consumers. Backyard and semi‐intensive poultry rearing is gaining popularity in Burkina Faso, but little attention has been paid to the potential negative impacts of zoonotic pathogens associated with poultry that can be transmitted to humans through poultry droppings. Infection with some of these pathogens, particularly *Salmonella* and/or *Campylobacter*, can have long‐term negative effects on the nutritional status of children due to the persistence of the infections they cause. For all these reasons, the goal of this project was to estimate the prevalence of *Salmonella* and *Campylobacter* in slaughtered poultry feces and carcasses sold in four markets of Ouagadougou.

## 2 MATERIAL AND METHODS

### 2.1 Study design

The study was carried from 5 November 2017 to 20 December 2017, and four chicken carcasses‐selling stalls at four large open markets located in low socioeconomic areas of Ouagadougou were enrolled. Prior to any other investigations, the aims of the study were explained to the chicken carcasses sellers. They were recruited into the study after the owners of the vending places had given their consent and the sellers were assured of confidentiality in accordance with the research protocol approved by the ethical committee. Observations of the working methods of the sellers and their stall surroundings were made, as well as of their hygienic practices, with instances of unhygienic behavior recorded. Completion of information was followed by a face‐to‐face interview in the local language.

### 2.2 Sampling

A total of 103 feces samples and 20 carcasses from slaughtered poultry were collected from the local poultry carcasses sellers in four retail markets of Ouagadougou, Burkina Faso. Immediately after slaughter, the whole intestine and/or carcass was collected aseptically using gloves after evisceration, placed in sterile plastic bags, and transported in a cool box to the laboratory. There were no records available about specific poultry farm locations, but according to poultry sellers, farms were located in different areas of the countryside.

### 2.3 *Salmonella* isolation

#### 2.3.1 Poultry carcasses processing

At the time of the experiment, whole carcasses were transferred into sterile plastic bags containing 400 ml of buffered peptone water (BPE; Liofilchem, Teramo, Italy). Bags were vigorously massaged and shaken for 1 min at room temperature. Rinse solutions were transferred to bottles and incubated at 37°C for 24 hr. After incubation, a 0.1 ml aliquot was transferred to 10 ml of Rappaport‐Vassiliadis Broth (RV: Oxoid, Basingstoke, UK) and incubated for 24 hr at 42°C. A loopful (10 μl) was then plated on xylose‐lysine‐deoxycholate agar (XLD; Oxoid) and incubated at 37°C for 24 hr.

#### 2.3.2 Poultry feces samples processing

Poultry feces samples were analyzed for *Salmonella* presence. One gram of the caeca contents was added to 9 ml of buffered peptone water, mixed, and incubated at 37°C for 24 hr. An aliquot of 0.1 ml was transferred to 10 mL of RV broth and incubated for 24 hr at 42°C. A loopful (10 μl) was then streaked on XLD agar and incubated at 37°C for 24 hr.

### 2.4 Identification of *Salmonella*

Colonies exhibiting typical Salmonella morphology on XLD agar plates were preliminarily confirmed biochemically using lysine iron and triple sugar iron agar slants as described in our previous study (Kagambèga et al., 2013). Final confirmation was made with API‐20E (Biomerieux, Marcy l'Etoile, France).

### 2.5 *Campylobacter* isolation

#### 2.5.1 Poultry carcasses processing

After transferring carcasses into a rinse solution, massaging, and shaking, as previously described, 10 ml was added into 10 ml of Bolton broth (Oxoid) supplemented with a selective supplement (SR0155 E; Oxoid). Samples were incubated at 42°C for 48 hr under microaerophilic conditions generated by a gas‐generating pack (Campygen CN25; Oxoid). Following incubation, a loopful of Bolton's broth was streaked onto modified cefoperazone charcoal deoxycholate agar plate (mCCDA; Oxoid) supplemented with a selective supplement (SR0155 E; Oxoid) and incubated at 42°C for 48 hr under microaerophilic conditions generated by a gas‐generating pack (Campygen CN25; Oxoid).

#### 2.5.2 Poultry feces samples processing

A loopful of the caeca contents was directly plated onto mCCDA agar plates supplemented with selective supplement (SR0155; Oxoid) and incubated at 42°C for 48 hr under microaerophilic conditions generated by a gas‐generating pack (Campygen CN25; Oxoid).

### 2.6 Identification of *Campylobacter*

Presumptive *Campylobacter*‐positive colonies on the mCCDA Agar plate were confirmed using the Gram's stain to observe the “corkscrew” morphology, the Campy‐latex agglutination test (Oxoid) and oxidase test (Hardy Diagnostics, Santa Maria, CA, USA). Confirmed colonies were further subjected to standard phenotypic tests using API CAMPY system (Biomerieux) to identify species level. The hippurate hydrolysis test was used to confirm speciation results.

## 3 RESULTS

### 3.1 Description of poultry transportation and carcasses vending conditions

**Figure 1:**
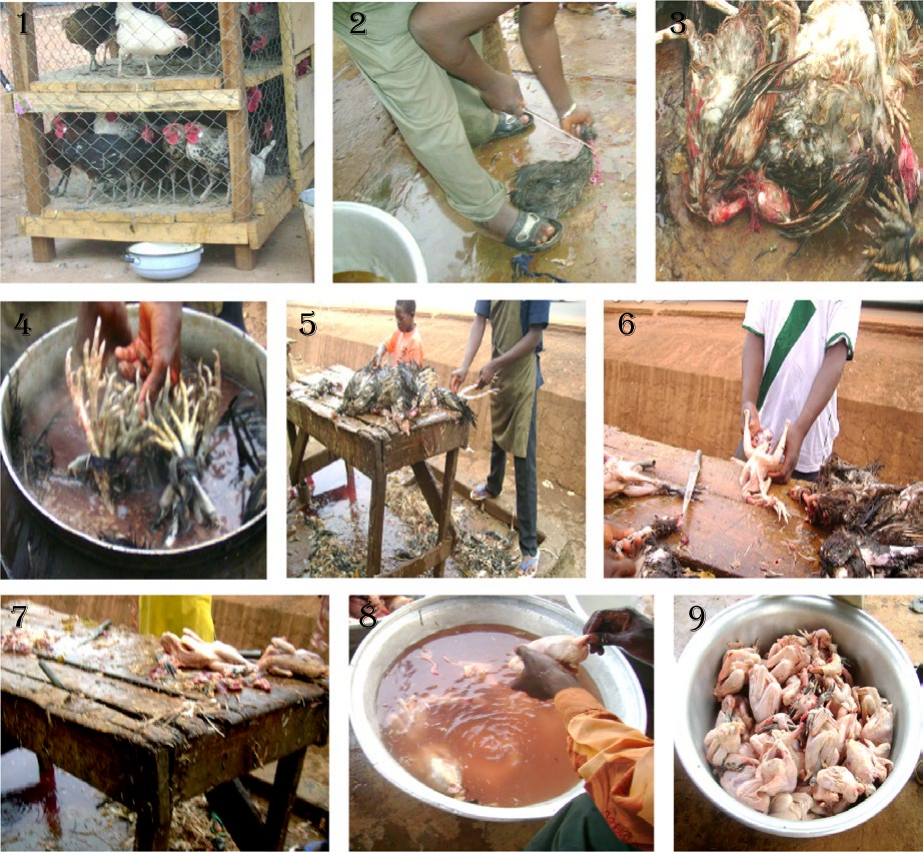
Typical poultry carcasses process followed by the vendors at the sampled markets. (1) Poultry vending site. (2) Killing step. (3) Draining step. (4)Scalding (dip of the chicken). (5) Plucking. (6) Evisceration. (7) Cleaning. (8) Rinsing. (9) Set for sale

Transportation systems such as bicycle, motorcycle, or car were used by vendors to transport their merchandise from different countryside farms to the markets. At the market, vendors kept chicken alive in railing before slaughter. The slaughter process was performed by the traditional slaughtering method at the market sites: Butchers killed by hand without gloves directly on the terrace. All the subsequent operations such as bleeding, plucking, evisceration, and cutting were executed on the same table. After that, poultry carcasses were rinsed in the same bucket of water and stored by hanging. Carcasses were sold off a table at ambient temperature without any type of protection from dust and flies at any point during the day (Figure 1).

The poultry vendors did not wear special clothes during poultry slaughtering or carcasses vending steps. In two of the four markets involved in this study, vendors were selling raw, grilled, and/or fried carcasses at the same place without separation. Their selling environment was infested by rodents, lizards, avian species, canine species, and/or feline species. After the market was closed, rodents, lizards, avian species, canine species, and/or feline species could be seen on or roaming around the vending tables, licking or sucking traces of blood or meat residues from the day. Potable water facilities were absent from the sampled markets. Therefore, on the next day of selling, tables were not cleaned with water or disinfectant before reception of new carcasses for sale.

### 3.2 Prevalence of *Salmonella* and *Campylobacter* in poultry feces and carcasses

Of the 103 fecal samples analyzed, 70 were positive for *Campylobacter* ssp (67.96%) and 54 for *Salmonella* ssp (52.42%) as shown in Table 1. The hippurate hydrolysis test revealed that among the 70 *Campylobacter* strains isolated from feces, 49 were *C. jejuni* (70%) and 21 were *C. coli* (30%). From the 20 carcasses analyzed, 18 were contaminated by *Salmonella* (90%) and 10 by *Campylobacter* ssp (50%) (Table 1). Among the 10 *Campylobacter* ssp isolated from poultry carcasses, all were identified as *C*. *jejuni* using the hippurate hydrolysis test. To complete information on unhygienic practices, from each site, chicken rinsing solution was taken two times and microbiological analysis revealed the presence of *Salmonella* spp in 100% (8/8) of these solutions.

**Table 1:** Prevalence of *Salmonella* and *Campylobacter* in poultry feces and carcasses samples collected in this study

Samples	*Salmonella* spp (%)	*Campylobacter* spp (%)	*Campylobacter jejuni* (%)	*Campylobacter coli* (%)
Feces (*n* = 103)	54 (52.42%)	70 (67.96%)	49 (70%)	21 (30%)
Carcasses (*n* = 20)	18 (90%)	10 (50%)	10 (100%)	0

## 4 DISCUSSION

The study revealed the poor hygienic practices of poultry carcasses sellers from different markets in Ouagadougou. These practices did not meet the hygiene levels for the handling of meat products as recommended by World Health Organization and the Food and Agriculture Organization *joint committee* (CAC, 2005). The hygienic status investigation revealed that all the poultry carcasses were rinsed in the same bucket of water after evisceration and sold off a table at ambient temperature without refrigeration or protection from dust and flies at any point during the day. These conditions certainly increase the risk of microbial contamination. Poor sanitation has been identified as the main contamination source in these environments. Moreover, insufficient personal hygiene, particularly among carcass handlers, contributed to the contamination of carcasses and the improper storage conditions favor microbial multiplication and, therefore, infections (Nkere, Ibe, & Iroegbu, 2011). These observations were also reported in previous studies: a food stored for a prolonged amount of time in an unsafe way will make an excellent growth medium (Beumer & Kusumaningram, 2003; Taulo et al., 2008).

In our study, we observed the presence of *Salmonella* spp in all the chicken‐washing solutions analyzed. The prevalence of *Salmonella* in poultry feces samples was 52.4% while 90% in carcasses. This difference in *Salmonella* prevalence between feces and carcasses could be explained by the fact that all carcasses are rinsed in same bucket of water. This step favors cross‐contamination between carcasses. In our previous study, where sampling was performed before the rinsing step, we reported only 57% of *Salmonella* presence in the poultry carcasses analyzed (Kagambèga et al., 2012). The prevalence of *Salmonella* in feces and carcasses found in this study was very high when compared to other studies. Moawad et al. (2017) reported recovery of *Salmonella* in 8.3% of the poultry carcasses in Egypt, and Odoch et al. (2017) reported *Salmonella* in 21.3% of the poultry feces in Uganda. A similar study in Senegal reported detection of non‐typhoidal *Salmonella* (NTS) in 35.1% of poultry fecal samples (Dione, Ieven, Garin, Marcotty, & Geerts, 2009). In Nigeria, Fagbamila et al. (2017) reported NTS in 43.6% of poultry feces. Among the above‐mentioned counties, Burkina Faso is the poorest nation. Chickens are not vaccinated against NTS, and in general, vaccinations for poultry are not mandatory. Therefore, the high prevalence of *Salmonella* in fecal samples is not surprising considering the operation of the poultry industry in Burkina Faso, where disease control efforts are poor and/or deficient.

The 90% *Salmonella* prevalence in carcasses reported in our study is also not unexpected considering the unhygienic conditions and practices observed at the vending sites. It is very likely that cross‐contamination during handling and preparation leads to carcasses contamination and Ingmer (2011) reported in their study that applying good cleaning and sanitization practices could effectively reduce *Salmonella* spp. on broilers.

No comparable data are available for *Campylobacter*, as this is the first study describing the presence of this pathogen in relation to the poultry industry in Burkina Faso. Our study revealed a significant prevalence of *Campylobacter* spp in poultry feces (67.96%) and in retail poultry carcasses (50%). These frequencies are consistent with those reported in previous studies conducted in Sri Lanka (Kottawatta et al., 2017) and in Ivory Coast (Goualié et al., 2012). Conversely, a higher prevalence of *Campylobacter* was observed in poultry carcasses in Japan (Furukawa et al., 2017), in Maryland (Cui, Ge, Zheng, & Meng, 2005), and in poultry feces in the Netherlands (Schets et al., 2017). Several factors might influence the prevalence of *Salmonella* and *Campylobacter* isolates in poultry meat: geographical location of farms, season in which the study was carried out, and differences in bacterial culture conditions and sampling methods (Williams & Oyarzabal, 2012).

*Campylobacter jejuni* and *C. coli* were the species isolated in this study. The isolation of these species from poultry is of public health importance, as these species of *Campylobacter* are known to cause infection in humans. *Campylobacter jejuni* and *C. coli* have been reported in patient with diarrhea in Burkina Faso (Bonkoungou et al., 2013). Poor hygiene and sanitation during the processing described in the present study could explain the high prevalence of *Campylobacter* on the carcasses.

Among the *Campylobacter* species identified, *C*. *jejuni* was the most prevalent with 70% rate in poultry feces and 100% in carcasses. Similar results were also reported in studies conducted in Ivory Coast (Goualié et al., 2012) and in Brazil (Perdoncini et al., 2015). In contrast, in Asia and Thailand, two studies revealed a higher percentage of *C. coli* than *C. jejuni* (Kottawatta et al., 2017; Padungtod & Kaneene, 2005). Nevertheless, our observations are concerning: Patients with diarrheal illnesses are not routinely sampled for *Campylobacter* in Burkina Faso, and poultry products are widely consumed.

In conclusion, we report a high prevalence of *Salmonella* and *Campylobacter* spp in poultry and poultry products in Burkina Faso. These results show a need to implement specific control procedures to decrease the contamination of poultry meat by *Salmonella* and *Campylobacter*. This study shows also the need to establish and/or implement a surveillance programs for foodborne pathogens. There is an urgent need to include *Campylobacter* detection in the bacteriological analysis of patient's samples.

The data from this study will allow the development of an effective national strategy for reducing enteric foodborne illness and will be an important tool for policymakers to assign and prioritize resources for food safety programs.

## CONFLICT OF INTEREST

The authors confirm that this article content has no conflict of interest.

## AUTHORS’ CONTRIBUTIONS

AK, DKS, FS, and PO carried out strain isolation. AK drafted the manuscript; AT and VT participated in writing the manuscript. PF and NB supervised the strain characterization and participated in writing the manuscript. All authors read, commented on, and approved of the final manuscript.

## ETHICAL REVIEW

Permission to conduct this study was obtained from poultry sellers. The study protocol was approved by the Ethical Committees of Burkina Faso.
